# A review of utilities and costs of treating upper extremity amputations with vascularized composite allotransplantation versus myoelectric prostheses in Canada

**DOI:** 10.1016/j.jpra.2022.03.003

**Published:** 2022-03-19

**Authors:** J.I. Efanov, B. Tchiloemba, A. Izadpanah, P.G. Harris, M.A. Danino

**Affiliations:** Plastic and Reconstructive Surgery Service, Department of Surgery, Centre Hospitalier de l'Université de Montréal, Montréal, Québec, Canada

**Keywords:** Upper extremity allotransplantation, Myoelectric prostheses, Utility outcomes study, Upper extremity amputation

## Abstract

**Background:**

Hand vascularized composite allotransplantation (VCA) and myoelectric prostheses have proven their efficacy for treating hand amputation. Despite reported functional outcomes, the lack of consensus on VCA versus myoelectric prostheses brought us to report on their utilities and costs within the Canadian healthcare system.

**Methods:**

A review of utility outcomes and costs was performed for VCA and myoelectric prostheses and a comparison between unilateral versus bilateral amputations was made.

**Results:**

The simulation model demonstrated that significant savings could be achieved with both hand transplantation ($10.04 billion) and myoelectric prostheses ($12.17 billion) in all Canadian patients sustaining hand amputation with a 30-year life expectancy., Myoelectric prosthesis had lowest total cost compared to hand VCA by generating savings of $4,458,445,840 and $1,868,121,840 when compared to bilateral and unilateral upper limb amputations respectively.

**Conclusion:**

Treatment of unilateral amputations with myoelectric prostheses would cost significantly less to the society, whereas the gap in cost savings becomes less significant in bilateral amputees. From the socioeconomic standpoint of the Canadian healthcare system, this simulation model demonstrates that significant savings can be achieved with both treatments.

## Introduction

Amputations of the upper extremity can undoubtedly be considered as one of the most devastating injuries one can suffer. Used for activities of daily living, the hand is an omnipresent and powerful tool, as well as an extension of a person's identity. The burden of living with an amputated hand can be reflected in several dimensions, such as function, psychological well-being, quality of life, cosmesis, cultural or spiritual associations, societal integration, and economic considerations.

Surgical and technological advancements have nonetheless allowed for hope in expanding the armamentarium of treatment options in the amputee population. Indeed, more than 180 vascularized composite allotransplantations (VCAs) of the hand have been performed worldwide in the past 20 years, a surgical prowess that restores like-with-like physically and functionally.^1^ However, complications due to lifelong immunosuppression is a major hurdle in acceptability of this experimental surgery as a gold-standard treatment.

Concurrently in the same timeframe as the advent of VCAs, engineers have refined pre-existing prosthetics to become “myoelectric-based”, whereby surface electrodes attached on the patient's intact proximal muscles could send signals to the device to produce specific movements with several degrees of liberty. Despite comparable functional outcomes with VCAs, myoelectric prostheses are plagued by high costs and high abandonment rates as well.^2^

Because of the low prevalence of these interventions and the lack of comparative studies, none can claim superiority of one treatment over the other at this time. Considering that both interventions require considerable costs to develop and implement, and that healthcare resources are increasingly limited, the debate between hand VCAs and myoelectric prostheses needs to include a discussion about health economics.

In this article, a review of utility outcomes and costs is performed for both interventions, with the objective to determine if hand amputees would benefit more from VCAs or myoelectric prostheses in a socioeconomic model with universal healthcare such as in Canada. A secondary objective is to compare unilateral versus bilateral amputees with respect to the cost-utility of both treatment groups.

## Methods

A review of the literature using the PRISMA guidelines was performed. Publication databases such as Embase, PubMed and Medline were employed for selection of articles. Different keywords were used for the different outcomes to be reported:

A) Prevalence of upper extremity amputations: “Prevalence”, “Incidence”, “Rate” or “Total number” AND “Upper extremity amputation”, “Hand amputation” or “Arm amputation”.
B) Costs of upper extremity amputations: “Costs”, “Financial loss” or “Economics” AND “Upper extremity amputation”, “Hand amputation” or “Arm amputation”.
C) Utilities of both interventions: “Utility”, “Utilities”, “QALY”, “Quality-adjusted life years”, “Cost-utility”, “Time trade-off” or “Standard gamble” AND “Hand transplantation”, “Upper extremity transplantation”, “Hand vascular composite allotransplantation”, “Upper extremity vascular composite allotransplantation”, “Myoelectric prosthesis” or “Externally-powered prosthesis”.
D) Costs of both interventions: “Costs”, “Economics” or “Financial” AND “Hand transplantation”, “Upper extremity transplantation”, “Hand vascular composite allotransplantation”, “Upper extremity vascular composite allotransplantation”, “Myoelectric prosthesis” or “Externally-powered prosthesis”.

Reference lists of each article were reviewed for relevance. Publications written in a language other than English were excluded. All articles pertaining to costs of either treatment within the Canadian healthcare system were reported separately. Finally, a simulation model was performed on the total costs of implementation of hand transplantation and myoelectric prostheses as nationalized programs. This simulation included potential gains from return to work and reported interventional costs multiplied by the prevalence of cases in Canada. Financial data was obtained from the Canadian governmental institution (www.statcan.qc.ca).

## Results

### Prevalence of upper extremity amputations

Few epidemiological studies quantify the problem, making it difficult to estimate the worldwide prevalence of individuals living with upper extremity amputations that are proximal to the wrist. Nonetheless, previous studies have postulated that there are more than 11.3 million unilateral amputees and 11.0 million bilateral amputees worldwide.^3^ However, these numbers reflect only traumatic causes of amputation and emanate from databases of high-income countries solely.

In Canada, no studies or governmental data were published specifically for hand amputees, but it is known that over 227,000 patients had suffered either a lower or upper extremity amputation as of 2013.^4^ By extrapolation from previous studies demonstrating that only 3% of limb amputees involve the upper extremity,^5^ a ballpark number of patients would be around 6800 Canadians amputated proximal to the wrist.

### Personal and societal costs of upper extremity amputations

Aside from functional and psychological consequences of living with a missing limb, upper extremity amputations provoke multifaceted repercussions from the economical and societal standpoints. In a pediatric population with major upper extremity amputations, the average length of hospital stay was 11.3 days, with a mean of 2.3 surgeries and hospitalization costs of $22,015 in 1996.^6^ Knowing that healthcare spending has grown by an average of 6% per year from 1993 to 2013,^7^ estimated hospital charges after an upper limb amputation could be around $59,281 in 2013.

A more recent study in 2018 found similar results, with mean hospital charges of $28,961 and a combined total cost of $166 million in the period from 1997 to 2012.^8^ These numbers are probably undervalued because the authors included finger amputations with proximal upper extremity amputations, which are significantly different in terms of costs to the healthcare system.

Furthermore, neither of these studies take into consideration any rehabilitative treatments such as prostheses and long-term care. Indeed, Blough et al. estimated that veterans with upper extremity amputation spend between $31,890 and $117,440 on average on prosthetics-related expenses over 5 years, depending on injury severity.^9^ Another study has postulated that direct healthcare costs over a lifetime can exceed $500,000 for an amputee.^10^

Another important consideration is the loss of revenue and the economic burden of social pension plans directed at upper extremity amputees. To help these patients, the *Canadian Pension Plan Disability Benefit* program^11^ offers an average monthly amount of $971,23, or $349,642 for a 30-year life expectancy.

Considering that limb loss to the upper extremity occurs at an average age of 42 years,^12^ an amputee in Canada will live almost half of his remaining life in this state (Canadian life expectancy at birth is 81.1 years, Statistics Canada 2017^13^). They will also be unable to contribute to the workforce for more than 22.5 years (average age at retirement in Canada is 64.5 years, Statistics Canada in 2020^14^) at a median yearly after-tax income of $62,900 (Statistics Canada in 2019^15^). This represents a loss of income production and taxes to the order of $1415,250 per amputee per lifetime if they don't return to work. Multiplied by an estimated 6800 individuals currently living with this disability in Canada, the total loss of revenue would be $9.6 billion per combined lifetimes.

### Utility studies in hand allotransplantation

Chung et al. was one of the first groups to publish data on the economic analysis of hand transplantation in 2010.^16^ They performed a cost-utility assessment with the “*time trade-off*” (TTO) technique on one hundred medical students. The authors reported that prosthetic use generated better cost-utility over unilateral hand VCA, but that bilateral hand transplants were favored over prosthetics. However, the “*incremental cost-utility ratio*” (ICUR) of double hand allotransplantation was $318 961/QALY, which exceeded the traditionally accepted threshold of $50,000/QALY^17^ (see Table 1).

**Table 1:** – Comparison of cost utility studies for upper extremity VCA.

Study	Study Groups	Transplantation health outcomes (QALY)/ (Health utility SG, TTO)	Standard of care health outcomes (QALY)/ (Health utility SG, TTO)	Transplantation Costs ($)	Standard of care costs: prosthetic ($)	Incremental cost-effectiveness ratio (ICER) in $
Health Quality Ontario, 2016	Unilateral amputation	10.96	11.82	735 647	61 429	*Dominated*
	Bilateral amputation	10.10	9.93	747 837	114 057	3 765 037
Alolabi et al., 2015	Hand Amputees	43.5/0.83(SG) 41.8/0.86(TTO)	35.7/0.70(SG) 34.9/0.69(TTO)	N/A	N/A	N/A
	General population	41.6/0.74 (SG) 35.7/0.82 (TTO)	40.7/0.72 (SG) 36.6/0.80 (TTO)	N/A	N/A	N/A
Brügger et al., 2010	Unilateral amputation	N/A	N/A	1 224 459 (*Lifetime cost*)	792 084 (*Lifetime cost*)	N/A
Chung et al., 2010	Unilateral amputation	28.81	30	528 293	20 653	*Dominated*
	Bilateral amputation	26.73	25.2	529 315	41 305	318 961

Another study investigating the utility analysis of hand transplantation was described by Alolabi et al. in 2015.^18^ The authors conducted QALY measurements in 30 participants from the general population and 12 amputees using the standard gamble (SG) and (TTO) techniques. They reported that the mean health utility of a hand amputation was 0.72 (SG) and 0.80 (TTO) for the general population as opposed to 0.69 (TTO) and 0.70 (SG) for amputees. In comparison, hand allotransplantation only slightly increased the utility in the general population to 0.74 (SG) and 0.82 (TTO), whereas it increased, albeit not significantly, in hand amputees to 0.83 (SG) and 0.86 (TTO). When translated into QALYs, patients with hand amputations reported increases of 7.0 (TTO) and 7.8 (SG) QALYs, as opposed to the general population with only a gain of 0.9 QALYs (See Table 1). However, the authors also reported that a decrease in life expectancy imparted a loss of 1.7 QALYs to the participants. Therefore, they concluded that there is no clear benefit to advocate for hand transplantation based on those results.

### Previous studies on *actual* costs of hand allotransplantation

Other studies have attempted to report *actual* costs of hand VCA. In a study by Brügger et al. from Switzerland, an economic cost-model of hand VCAs was performed.^19^ The study found that, for a model patient of 30 years old, with a unilateral forearm amputation, undergoing hand allotransplantation at 35 years old, with a remaining life expectancy of 46.1 years, the lifetime cost would be $1224,459. In comparison, the authors found that the lifetime cost for the same patient treated with a conventional prosthesis would be $792,084, a difference of $432,374.

In the same study described earlier from Chung et al., the authors stipulate that the actual surgical cost for a unilateral and bilateral hand transplantation would be $18,351 and $19,432 CAD, which includes preoperative evaluation, hospitalization, and physician fees.^20^ These values were estimated from CPT codes of forearm replantation but failed to account for several variables. The majority of remaining costs actually originates from lifetime immunosuppressive drugs and clinic visits, estimated at $576,405.

### Costs of performing hand vca in canada

When major decisions affecting the health of Canadians are made, the *Canadian Agency for Drugs and Technologies in Health* (CADTH) requires that investigators provide a solid foundation of evidence-based medicine on the topic. One model proposed to conduct these endeavours is a *Health Technology Assessment* (HTA)” .^21^

Prior to performing this technique for the first time in Canada, the provincial advisor on the quality of health care in Ontario (*Health Quality Ontario*) completed a health technology assessment.^4^ In their results, they report that, for a healthy adult with a 30-year lifespan, a unilateral hand allotransplantation would cost $735,647 as opposed to $747,837 for bilateral transplantation, both significantly more expensive than the standard of care (defined as no transplantation) calculated at $61,429 and $114,057 for unilateral and bilateral hand amputation respectively. When analysing quality-adjusted life-years, single-hand transplants failed to supplant standard of care (10.96 versus 11.82), whereas double-hand transplants increased effectiveness by 0.17 QALYs only (10.10 versus 9.93).

The authors also calculated the “*incremental cost-effectiveness ratio”* (ICER), which conveys how much money it would cost to gain 1 QALY from that procedure. The value was estimated to be $3.8 million per QALY, which far exceeds the accepted threshold of $66,516 per additional QALY reported in the scientific literature on kidney transplants.^22^ The *Health Quality Ontario* group further postulated that a hand transplantation program would require a budget of $0.9–1.2 million over 3 years to treat 3 patients per year. They concluded that due to the low quality of evidence with respect to the benefits of upper extremity VCAs, both unilateral and bilateral hand allotransplantation were not cost-effective in comparison with standard of care.

Several limitations of this study on hand transplantation HTA need to be underlined. First, the calculations of QALYs on which the incremental cost-utility ratios are calculated are derived from the study by Chung et al. in 2010.^23^ Considering the absence of other comparative QALY measures on hand transplant recipients and the lack of calculation of QALYs on the population represented by Health Quality Ontario, the conclusions emanating from this study suffer from low evidence. Second, the comparative group labeled as standard of care refers to patients not receiving any sort of treatment. One could argue that the current standard of care is in fact myoelectric prosthetics. The cost-utility assessment could be different if only patients with prosthetics were included in the analysis. Third, several costs were ignored or underestimated, such as the cost of rehabilitation after 2 years, the number of personnel involved in preoperative, perioperative, and postoperative care and the management of more than two major complications or any minor complication in the early and late post-transplantation phase.

### Utility studies with myoelectric prostheses

Few studies reported on the utility outcome measures, the quality-adjusted life years and the cost-utility analyses specifically associated with myoelectric prostheses.

One study looking at utilities in myoelectric prosthetics was published as a conference abstract by Baltzer et al. in 2018,^24^ whereby a Markov decision analytic model was used to determine the cost-utility of VCA, myoelectric prostheses, and body-powered prostheses for transradial amputations. The authors found body-powered prosthetics to be the most cost-effective option, which increased QALYs by 14.45 at a cost of $281,795 over a lifetime (ICUR of $19,501/QALY, corresponding to the acceptability threshold of less than $50,000/QALY). In comparison, myoelectric prostheses, and upper extremity VCA produced ICURs of $75,895/QALY and $780,061/QALY, respectively. However, when myoelectric devices cost less than $31,000, they became the preferred strategy of treatment. Although interesting, these results cannot be critically appraised due to the lack of methodological details provided in the conference abstract.

Our group was the first to publish utility health outcomes and QALYs associated with myoelectric prostheses in unilateral and bilateral amputees.^25^ Patients with a bilateral amputation reported utility outcomes of 0.71 (TTO) and 0.68 (SG) in the scenario of receiving a myoelectric prosthesis with no complications, and 0.52 (TTO) and 0.50 (SG) with complications. On the other hand, patients with a unilateral amputation demonstrated an increase in utility measures for the scenario with no complications (0.84 TTO and 0.81 SG) but a decrease when there were associated complications (0.45 TTO and 0.42 SG). When calculated as QALYs, patients with unilateral amputations and those who had sustained a replantation procedure reported significantly higher QALYs for myoelectric prostheses than hand VCA (+6.4, *p* = 0.0015 and +8.4, *p* = 0.0001 respectively).

### Previous studies on *actual* costs of myoelectric prostheses

The literature review on actual costs of myoelectric prostheses is difficult to interpret because of the large variety of devices available on the market, the different techniques based on the level of amputation, and the need for associated surgical procedures such as “*targeted muscle reinnervation*” (TMR).

Costs of commercially available myoelectric devices encompass a wide range, with one study suggesting that it can vary between $20,000 to $100,000.^26^ In a whitepaper published at the *Bioengineering Institute Center for Neuroprosthetics*, the authors report that the typical cost varies by level of amputation, with cosmetically realistic hands at $20,000-$30,000 versus full-arm neuroprosthetics at $100,000.^26^

Another study by Resnik et al. also reports similar ranges of pricing based on the *New York region Centers for Medicare and Medicaid Services*.^27^ Transradial and transhumeral externally powered devices cost $25,000-$50,000 and $50,000-$75,000, respectively. This corresponds to a five-fold increase in costs when compared with body powered prosthetics in the same study.

Similarly, the *Department of Veterans Affairs* in the United States reports that a myoelectric device would cost a mean of $18,703 for partial hands, $20,329 for transradial, $59,664 for transhumeral, $61,655 for shoulder and $62,271 for forequarter disarticulation.^28^ Additionally, the same group demonstrated with a Markov model that the 5-year projected cost for a unilateral amputee would be $31,129 to $117,440, whereas multiple limbs would increase the cost to $130,890 to $453,696. Over a lifetime model, the mean cost was calculated at $823,239.

### Costs of implementing myoelectric prostheses in canada

Only one study has been published in Canada with respect to costs of owning an upper extremity myoelectric prosthesis.^29^ In their work, Chan et al. retrospectively reviewed 28 amputees who had received prosthetics, either body-powered or myoelectric. Over a five-year period, the total cumulative cost to the healthcare system was $65,520 per patient. The most substantial portion of these costs occurred during the first year which includes the cost of the device, the fitting, and the intensive rehabilitation. The average annual cost was significantly higher, as expected, in myoelectric prostheses when compared to body-powered.

They report a mean number of prosthetics repair of 1.64 per year, which is more significant in transradial amputees (1.96/year) than transhumeral amputees (1.26/year) .^29^ There was no significant difference in number of repairs between body-powered and myoelectric prostheses, except for transradial devices whereby myoelectric technology required twice as many repairs (1.39/year versus 0.78/year). Aside from repairs, an upper limb amputee needs to make device adjustments every two years (0.49/year average).

Rates of abandonment also need to be considered in the total cost calculation. Out of 20 patients who had a follow-up of more than 3 years, 12 have not been using their initial prosthesis, including 8 body-powered and 4 myoelectric. The authors considered that the costs of these abandoned prostheses that could have been saved amounted to $305,922.

### Gains from potential return to work

When analysing the cost-benefit of these interventions, the potential gains obtained from return to work need to be included in the analysis. This information is sparse in the literature for hand VCAs and myoelectric prostheses. Only one report demonstrated return to work in 8 out of 12 (66%) transplant recipients.^30^ As for myoelectric prostheses, studies report rates of return to work at 80%.^31^

**Fig. 1:**
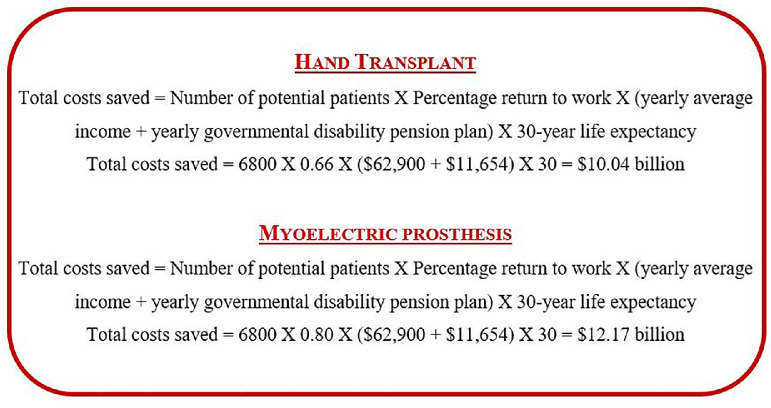
Potential total cost savings in the Canadian population if upper limb amputees underwent VCA or myoelectric prostheses over a 30-year lifespan.

If we assume that 66% of hand transplant recipients would return to work, the total savings in terms of loss of income and disability pension plans for 6800 upper limb amputees in Canada would be $10.04 billion for a 30-year lifespan (Fig. 1). In comparison, with the assumption of an 80% rate of return to work, myoelectric prostheses would save $12.17 billion for the same 30-year lifespan in the same population.

### Cost comparisons between hand vca and myoelectric prostheses

**Fig. 2:**
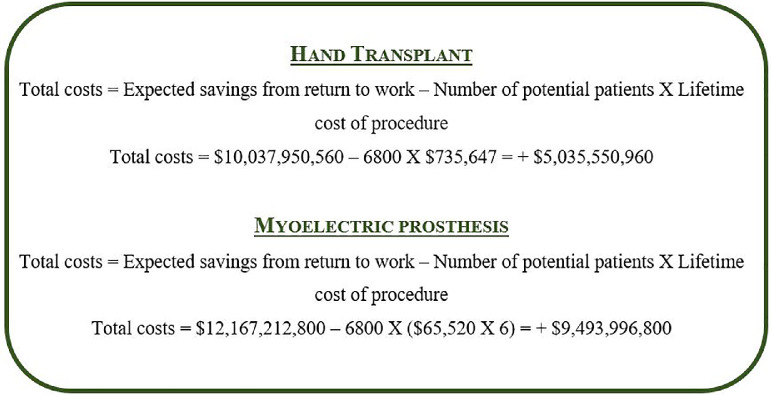
Total costs of implementing VCAs or myoelectric prostheses in all Canadian patients with *unilateral* upper limb amputations subtracted from expected savings from return to work over a 30-year lifespan.

**Fig. 3:**
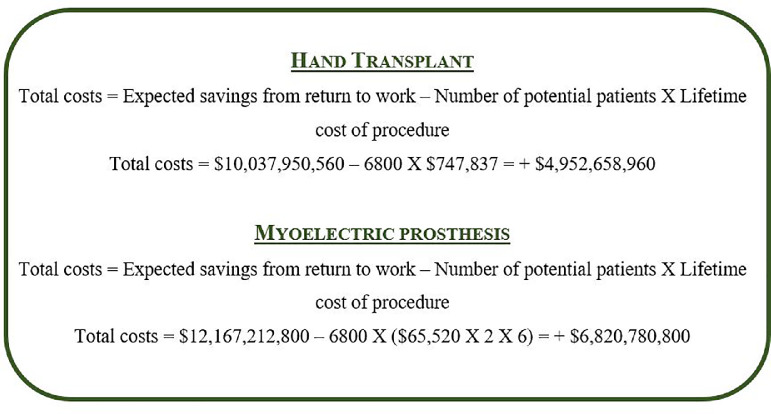
Total costs of implementing VCAs or myoelectric prostheses in all Canadian patients with *bilateral* upper limb amputations subtracted from expected savings from return to work over a 30-year lifespan.

Using the data reviewed in this article, Fig. 2 and Fig. 3 demonstrate simulation models of how much it would cost for the Canadian healthcare to treat all patients with either intervention and subtracting how much would be saved from return to work in unilateral and bilateral amputees. The total number of Canadian amputees who would benefit from either intervention (*n* = 6800) is obtained from the calculation that 3% of all limb amputations are upper limbs and that there are an estimated 227,000 amputees in Canada.^4^ The total lifetime cost of a unilateral ($735,647) and a bilateral ($747,837) upper extremity VCA was obtained from the study published by *Health Quality Ontario*.^4^ The total lifetime cost of a myoelectric prosthesis was calculated from the study published by Chan et al., approximating $65,520 for a 5-year lifespan.^29^ This amount was multiplied by a factor of 6 in order to determine the cost over a lifespan of 30 years, and by an additional factor of 2 in the case of bilateral myoelectric prostheses.

## Discussion

To date, this is the first study comparing lifetime costs of upper extremity VCA and myoelectric prostheses.

Several limitations of this simulation model need to be addressed. First of all, it is unrealistic to suppose that all amputees would be candidates for VCA or myoelectric prostheses. Indeed, contra-indications for allotransplantation, and to a lesser extent for myoelectric prosthetics, are very stringent, such as a lack of cognitive capacity in understanding the risks and benefits, a demonstration of lack of compliance with subsequent rehabilitation, any un-addressed psychological disorders that would impair long-term success, active infection/malignancy, congenital limb absence, and more.^32^ Although savings in the order of billions of dollars can be accomplished if all patients were treated with one of these interventions, even a smaller percentage of eligible candidates would produce significant benefits. For example, if as little as 5% of all 6800 Canadian amputees were *actual* candidates, the expected benefits would be $250 million for hand VCA and $474 million for myoelectric prostheses.

Second of all, extrapolation of lifetime costs from the aforementioned studies^4^ is probably an under-estimation of the real costs. For allotransplantation, the values used come from analytic models that did not include all associated costs related to long-term complications of immunosuppression, because the rates of these complications are still unknown. For myoelectric prostheses, the study used to make the calculations only considered the cost of the device and its maintenance but failed to include costs of rehabilitation and costs of potential complications.

Third of all, although rates of return to work have been described in the literature, none of these studies report if patients actually return to their pre-amputation work or if they require career reorientations. The expected lifetime savings from return to work are based on average income of all Canadians, which is not necessarily the same as the expected outcomes that these patients would gain if they returned to lower-pay salaries.

Finally, the total cost savings from return to work are calculated over a lifetime of 30 years. Even though most patients suffering an amputation are in their working years (average age 42 years^33^), a significant portion would not be capable to return to the workforce because of retirement age, therefore nullifying the potential monetary gains of undergoing an allotransplantation or a myoelectric prosthesis.

## Conclusion

Although no economical model can be perfect, this article presented a review of all studies pertaining to the measurement of utility outcomes, QALYs and cost-utility analyses of upper extremity allotransplantation and myoelectric prostheses. Based on the currently available data, no consensus can be made to affirm that one procedure is superior to the other and should therefore be considered the “gold-standard”. However, most utility studies demonstrate that unilateral amputees have superior outcomes with myoelectric prostheses. On the other hand, bilateral amputees continue to be adequate candidates for both interventions.

From the socioeconomic standpoint of the Canadian healthcare system, our simulation model demonstrates that significant savings can be achieved with both treatments. Here again, treatment of unilateral amputations with myoelectric prostheses would cost significantly less to the society, whereas the gap in cost savings becomes less significant in bilateral amputees. In either case, implementing these treatment programs will not be an inexpensive endeavor to accomplish.

## Declaration of Competing Interest

None declared.

## References

[1] Kinsley S.E., Lenhard N.K., Lape E.C. (2021 Aug). Perceived Success in Upper-Extremity Vascularized Composite Allotransplantation: a Qualitative Study. J Hand Surg Am.

[2] Biddiss E.A., Chau T.T. (2007). Upper limb prosthesis use and abandonment: a survey of the last 25 years. Prosthet Orthot Int.

[3] McDonald C.L., Westcott-McCoy S., Weaver M.R., Haagsma J., Kartin D. (2021 Apr 1). Global prevalence of traumatic non-fatal limb amputation. Prosthet Orthot Int.

[4] Health Quality Ontario (2016 Jun 1). Composite Tissue Transplant of Hand or Arm: a Health Technology Assessment. Ont Health Technol Assess Ser.

[5] Ziegler-Graham K., MacKenzie E.J., Ephraim P.L., Travison T.G., Brookmeyer R. (2008 Mar). Estimating the prevalence of limb loss in the United States: 2005 to 2050. Arch Phys Med Rehabil.

[6] Trautwein L.C., Smith D.G., Rivara F.P. (1996 Nov). Pediatric amputation injuries: etiology, cost, and outcome. J Trauma.

[7] https://www.thebalance.com/causes-of-rising-healthcare-costs-4064878.

[8] Vakhshori V., Bouz G.J., Mayfield C.K., RK Alluri, Stevanovic M., Ghiassi A. (2019 Nov). Trends in Pediatric Traumatic Upper Extremity Amputations. Hand (N Y).

[9] Blough D.K., Hubbard S., McFarland L.V., Smith D.G., Gambel J.M., Reiber G.E. (2010). Prosthetic cost projections for servicemembers with major limb loss from Vietnam and OIF/OEF. J Rehabil Res Dev.

[10] MacKenzie E.J., Jones A.S., Bosse M.J. (2007 Aug). Health-care costs associated with amputation or reconstruction of a limb-threatening injury. J Bone Joint Surg Am.

[11] CanadaGo. Canada Pension Plan Disability Benefit – How much could you receive https://www.canada.ca/en/services/benefits/publicpensions/cpp/cpp-disability-benefit/benefit-amount.html 2018. Accessed September 3rd 2021

[12] Inkellis E., Low E.E., Langhammer C. (2018 Apr 24). Morshed S. Incidence and Characterization of Major Upper-Extremity Amputations in the National Trauma Data Bank. JB JS Open Access.

[13] Canada S. Archived - Life expectancy at birth and at age 65, by province and territory, three-year average. 2017; https://www12.statcan.gc.ca/census-recensement/2016/dp-pd/prof/details/page.cfm?Lang=E&Geo1=CSD&Code1=2466023&Geo2=PR&Code2=24&SearchText=Montreal&SearchType=Begins&SearchPR=01&B1=All&TABID=1&type=1. Accessed September 3rd 2021.

[14] Canada S. Retirement age by class of worker. 2021; https://www150.statcan.gc.ca/t1/tbl1/en/tv.action?pid=1410006001. Accessed September 3rd 2021.

[15] Canada S. Canadian Income Survey. 2019; https://www150.statcan.gc.ca/n1/daily-quotidien/210323/dq210323a-eng.htm. Accessed September 3rd 2021

[16] Chung K.C., Oda T., Saddawi-Konefka D., Shauver M.J. (2010). An economic analysis of hand transplantation in the United States. Plast. Reconstr. Surg..

[17] Braithwaite R.S., Meltzer D.O., King J.T., Leslie D., Roberts M.S (2008). What does the value of modern medicine say about the $50,000 per quality-adjusted life-year decision rule?. Med Care.

[18] Alolabi N., Chuback J., Grad S., Thoma A. (2015). The utility of hand transplantation in hand amputee patients. J Hand Surg Am.

[19] Brugger U., Plessow R., Hess S., Caballero A., Eichler K., Meyer V. (2015). The health technology assessment of the compulsory accident insurance scheme of hand transplantation in Switzerland. The Journal of hand surgery, European volume..

[20] Chung K.C., Oda T., Saddawi-Konefka D., Shauver M.J. (2010). An economic analysis of hand transplantation in the United States. Plast. Reconstr. Surg..

[21] CADTH. About the Health Technology Assessment Service https://www.cadth.ca/about-cadth/what-we-do/products-services/hta2018. Accessed September 3rd 2021

[22] Braithwaite R.S., Meltzer D.O., King J.T., Leslie D., Roberts M.S (2008). What does the value of modern medicine say about the $50,000 per quality-adjusted life-year decision rule?. Med Care.

[23] Chung K.C., Oda T., Saddawi-Konefka D., Shauver M.J. (2010). An economic analysis of hand transplantation in the United States. Plast. Reconstr. Surg..

[24] Robbins N.L., Wordsworth M.J., Parida B.K. (2019). A Flow Dynamic Rationale for Accelerated Vascularized Composite Allotransplant Rejection. Plast Reconstr Surg.

[25] Efanov J.I., Izadpanah A., Bou-Merhi J., Lin S.J., Danino M.A. (2022 Mar 1). Applying Health Utility Outcome Measures and Quality-Adjusted Life-Years to Compare Hand Allotransplantation and Myoelectric Prostheses for Upper Extremity Amputations. Plast Reconstr Surg.

[26] McGimpsey B. Limb Prosthetics Services and Devices Critical Unmet Need: market Analysis. 2017; https://www.nist.gov/system/files/documents/2017/04/28/239_limb_prosthetics_services_devices.pdf. Accessed September 3rd 2021

[27] Resnik L., Meucci M.R., Lieberman-Klinger S. (2012 Apr). Advanced upper limb prosthetic devices: implications for upper limb prosthetic rehabilitation. Arch Phys Med Rehabil.

[28] Blough D.K., Hubbard S., McFarland L.V., Smith D.G., Gambel J.M., Reiber G.E. (2010). Prosthetic cost projections for servicemembers with major limb loss from Vietnam and OIF/OEF. J Rehabil Res Dev..

[29] Chan A., Kwok E., Bhuanantanondh P. (May 2013). Cost of Ownership of Upper Limb Prostheses: a Retrospective Analysis. CMBES Proc.

[30] Petruzzo P., Lanzetta M., Dubernard J.M., Margreiter R., Schuind F., Breidenbach W. (2008). The international registry on hand and composite tissue transplantation. Transplantation.

[31] Fernández A., Isusi I., Gómez M. (2000 Aug). Factors conditioning the return to work of upper limb amputees in Asturias, Spain. Prosthet Orthot Int.

[32] Hollenbeck S.T., Erdmann D., Levin L.S. (2009 Mar). Current indications for hand and face allotransplantation. Transplant Proc.

[33] Inkellis E., Low E.E., Langhammer C. (2018 Apr 24). Morshed S. Incidence and Characterization of Major Upper-Extremity Amputations in the National Trauma Data Bank. JB JS Open Access.

